# Relationship between systolic blood pressure and mortality in older *vs* younger trauma patients – a retrospective multicentre observational study

**DOI:** 10.1186/s12873-023-00863-1

**Published:** 2023-09-19

**Authors:** Axel Benhamed, Brice Batomen, Valérie Boucher, Krishan Yadav, Chartelin Jean Isaac, Eric Mercier, Francis Bernard, Julien Blais-L’écuyer, Karim Tazarourte, Marcel Emond

**Affiliations:** 1 CHU de Québec-Université Laval Research Centre, Québec, Québec Canada; 2 Département de Médecine Familiale et de Médecine d’urgence, Université Laval, Québec, Québec Canada; 3 Hospices Civils de Lyon, Service d’Accueil des Urgences – SAMU 69, Centre Hospitalier Universitaire Edouard Herriot, Lyon, 69003 France; 4 Dalla Lana School of Public Health, University of Toronto, Toronto, ON Canada; 5 Department of Emergency Medicine, University of Ottawa, Ottawa, ON Canada; 6 Ottawa Hospital Research Institute, Ottawa, ON Canada; 7 Critical Care Unit, Hopital du Sacre-Coeur de Montreal, Montreal, QC Canada; 8 Research on Healthcare Performance (RESHAPE), INSERM U1290, Université Claude Bernard Lyon 1, Lyon, 69003 France

**Keywords:** Blood pressure, Trauma, Older patients, Triage, Mortality

## Abstract

**Background:**

The population of older trauma patients is increasing. Those patients have heterogeneous presentations and need senior-friendly triaging tools. Systolic blood pressure (SBP) is commonly used to assess injury severity, and some authors advocated adjusting SBP threshold for older patients. We aimed to describe and compare the relationship between mortality and SBP in older trauma patients and their younger counterparts.

**Methods:**

We included patients admitted to three level-I trauma centres and performed logistic regressions with age and SBP to obtain mortality curves. Multivariable Logistic regressions were performed to measure the association between age and mortality at different SBP ranges. Subgroup analyses were conducted for major trauma and severe traumatic brain injury admissions.

**Results:**

A total of 47,661 patients were included, among which 12.9% were aged 65–74 years and 27.3% were ≥ 75 years. Overall mortality rates were 3.9%, 8.1%, and 11.7% in the groups aged 16–64, 65–74, and ≥ 75 years, respectively. The relationship between prehospital SBP and mortality was nonlinear (U-shape), mortality increased with each 10 mmHg SBP decrement from 130 to 50 mmHg and each 10-mmHg increment from 150 to 220 mmHg across all age groups. Older patients were at higher odd for mortality in all ranges of SBP. The highest OR in patients aged 65–74 years was 3.67 [95% CI: 2.08–6.45] in the 90–99 mmHg SBP range and 7.92 [95% CI: 5.13–12.23] for those aged ≥ 75 years in the 100–109 mmHg SBP range.

**Conclusion:**

The relationship between SBP and mortality is nonlinear, regardless of trauma severity and age. Older age was associated with a higher odd of mortality at all SBP points. Future triage tools should therefore consider SBP as a continuous rather than a dichotomized predictor.

## Background

As life expectancy is increasing, trauma patients’ demographic is rapidly evolving. Since 2014, older adults aged 65 years and over represent the majority of admissions in Level-I trauma centres in the province of Québec (Canada) [1]. In the United Kingdom, a substantial increase (from 8.1% in 1990 to 26.9% in 2013) of the proportion of adults aged 75 years and over sustaining major trauma was reported [2]. In the USA, the proportion of older (≥ 65 years) trauma patients increased from 18 to 30% between 2005 and 2015 [3]. The aging trauma population represents a worldwide challenge for clinicians because of heterogeneous patient presentations due to frailty [4], pre-existing comorbidities [5], and the use of medications such as beta-blockers or anticoagulants [6] that alter physiological responses to trauma. Furthermore, advanced age has been associated with undertriage (inaccurate triage that results in a patient who requires higher-level care not being transported to a Level I or Level II trauma centre) [7], longer length of stay [8] and poor outcomes (higher mortality, morbidity and adverse events) compared to younger adults [9].

However, recent systematic reviews reported that current prehospital trauma triage tools may not accurately identify older patients with major trauma [10, 11]. It is therefore imperative to adapt trauma care with senior-friendly tools. For example, using a specific geriatric protocol to identify high-risk patients was found to reduce mortality [12]. Elsewhere, modified triage tools have been evaluated and have shown better sensitivity in identifying major trauma patients who need specialized trauma care [11, 13]. Although trauma patients’ initial systolic blood pressure (SBP) is commonly used as a severity and mortality indicator in triage protocols and as a trauma team activation criterion the standard SBP threshold (< 90 mmHg) may need to be adjusted for older patients [14, 15] or higher [16] could be a sign of hypotension and that this threshold could better identify seriously injured older patients [17]. However, the literature is heterogenous, and there is no consensus on whether it is accurate to use a unique threshold to define hypotension in the population of trauma patients.

Therefore, the main objective of this study was to describe and compare the relationship between mortality and systolic blood pressure (SBP) in older patients (65–74 years and ≥ 75 years) and their younger counterparts (16–64 years).

## Methods

### Study design and setting

We conducted a multicentre retrospective study based on the provincial Quebec Trauma Registry (RTQ), managed by the Quebec Ministry of Health and Social Services (*Ministère de la Santé et des Services sociaux*). The RTQ includes data from the 59 designated trauma centres of the province’s inclusive trauma system, which serves a population of 8.5 million in a geographic area of approximately 1.7 million km^2^ [18]. The system includes three Level-I adult trauma centres and two paediatric trauma centres providing highly specialized care in metropolitan areas (Montréal and Québec city). Trauma level designations are based on American College of Surgeons’ criteria and are periodically revised following on-site visits [19, 20].

### Study population

We included patients aged ≥ 16 years with a primary diagnosis of trauma between March 2003 and December 2017 who were directly transported from the prehospital setting or transferred to one of the three level-I adult trauma centres. We excluded patients aged ≥ 65 years admitted for isolated hip fractures since this type of trauma is often related to chronic disease rather than a traumatic event [21]. Patients were stratified into three age groups: 16–64, 65–74, and ≥ 75 years.

### Study data

Data from this provincial registry is prospectively collected and coded by trained medical archivists using patient electronic medical charts [22]. The RTQ is centralized and subject to systematic and periodic validation by ministry delegates to identify and rectify aberrant data values and verify date and time chronology. Inter-rater reliability is assessed randomly, thus ensuring a 98% accuracy.

Initial SBP was the first SBP measured by paramedics in the prehospital setting. We then used the first SBP measured upon ED admission if not available. We used the SBP measured in the initial ED if prehospital SBP was unavailable in transferred patients. The Injury Severity Score (ISS) was calculated after anatomic assessments were completed and was based on the 2008 version of the Abbreviated Injury Scale (AIS).

### Outcome measures

The primary outcome of this study was in-hospital mortality (including ED deaths).

### Data analysis

Categorical variables were described with frequencies and percentages, while medians with interquartile range (IQR), means, and standard deviation (SD) were computed for continuous variables. To obtain mortality curves by prehospital or ED SBP, we performed logistic regressions with age groups, and SBP was modelled using restricted cubic splines with 4 knots placed at the (5-35-65-95) percentiles. Knot locations were identified heuristically using Harrel’s Regression Modelling Strategies, and 95% CI were obtained using a clustered bootstrap [23]. The inflexion points of each curve were determined using the finite difference formula [24]. Three time periods (2003–2007, 2008–2012, and 2013–2017) were defined to assess whether there was a cohort effect. Odds ratios were computed to determine the association between age and mortality at different ranges of SBP, controlling for the following covariates: sex, mechanism, ISS, and comorbidities. We calculated sensitivity, specificity, and positive and negative predictive values for each SBP cutoff per 10 mmHg from 90 to 130 mmHg. We used multiple imputation with chained equation to handle missing Glasgow Coma Scale (GCS) score data [25]. We used Rubin’s rules to combine estimates across imputed datasets and to obtain 95% Confidence Intervals (95% CI) [26]. Patients with severe Traumatic Brain Injury (TBI, GCS score ≤ 8) and major trauma (ISS > 12) [27] present unique challenges and complexities compared to the broader population of trauma patients. This may be due to the distinct clinical characteristics, treatment needs, and potential variations in the relationship between SBP and mortality within these subgroups. We therefore performed further analyses within these two specific patient populations. Statistical analyses were conducted using SAS (Statistical Analysis System v9.4, SAS Institute Inc., Cary, NC, USA).

### Ethics approval

The CHU de Québec-Université Laval Research Ethics Board approved this project (MP-20-2017-3180).

## Results

### Sociodemographic characteristics

Overall, 53,324 trauma admissions were recorded within the three level-I adult trauma centres. Of those, 47,661 met our inclusion criteria and were therefore considered for analyses. Patients aged 16–64 years represented 59.8% of our cohort, 12.9% were aged 65–74 years, and 27.3% were 75 years and over. The most common mechanism of trauma was fall in both groups of older patients (65–74 years: 73.1%, and ≥ 75 years: 86.6%), while motor vehicle collision was the most common among younger adults (38.5%). Mean ± SD SBP was higher in older patients (65–74 years: 141.8 mmHg ± 27.3, and ≥ 75 years:147.9 mmHg ± 27.6) compared to younger patients (131.3 mmHg ± 23.5). Mean ± SD ISS was 14 ± 11 in patients under the age of 65 and 14 ± 10 in patients aged 65–74 years, while it decreased to 12 ± 8.8 in those aged 75 years and over. In-hospital mortality was higher in older patients (3.9%, 8.1% and 11.7% for those aged 16–64 years, 65–74 years and ≥ 75 years, respectively, Table 1). The median [IQR] time between trauma and death was 5.3 [1.9–14.3] days.

**Table 1:** Baseline patient characteristics

**Epidemiology**	** < 65 years** *n* = 28,502 (59.8%)	**65–74 years** *n* = 6,156 (12.9%)	** ≥ 75 years** *n* = 13,003 (27.3%)	**Total** *n* = 47,661
	n (%)	n (%)	n (%)	n (%)
Age, years, mean ± SD	40.8 ± 14.6	69.6 ± 2.9	83.6 ± 5.7	56.2 ± 22.5
Sex, male	20,794 (73.0)	3,430 (55.7)	4,936 (38.0)	29,200 (61.3)
Comorbidities, ≥ 1	8,870 (31.1)	4,432 (55.8)	8,940 (69.8)	21,242 (45.6)
Missing data	3,228 (11.3)	379 (6.2)	517 (4.0)	4,134 (8.7)
Transfer from another hospital	7,624 (26.7)	1,716 (27.9)	2,452 (18.9)	11,792 (24.7)
**Trauma mechanism**				
Motor vehicle collision	10,984 (38.5)	1,117 (18.1)	1217 (9.4)	13,318 (27.9)
Fall	10,787 (37.9)	4,497 (73.1)	11,264 (86.6)	26,548 (55.7)
Penetrating	2,336 (8.2)	110 (1.8)	69 (0.5)	2,515 (5.3)
Other	4,395 (15.4)	432 (7.0)	453 (3.5)	5,280 (11.1)
**Emergency department clinical assessment**				
Systolic blood pressure, mmHg	131.3 (23.5)	141.8 (27.3)	147.9 (27.6)	136.6 (26.9)
Missing data	741 (1.6)	90 (0.2)	140 (0.3)	981 (2.1)
Glasgow coma scale				
13–15	12,518 (43.9)	2,891 (47.0)	6,714 (51.6)	22,123 (46.4)
9–12	914 (3.2)	163 (2.6)	357 (2.7)	1,434 (3)
≤ 8	2,194 (7.7)	380 (6.2)	636 (4.9)	3,210 (6.8)
Missing data	12,876 (45.2)	2,722 (44.2)	5,296 (40.7)	20,894 (43.8)
Severe TBI, n (%)	1303 (2.73)	212 (0.44)	270 (0.56)	1785 (3.74)
Injury severity score, median [IQR]	10 [5–20]	9 [5–20]	9 [4–16]	9 [5–18]
> 12	12,768 (44.8)	2,718 (44.1)	4,381 (33.7)	19,867 (41,68)
Missing data	39 (0.1)	5 (0.1)	0 (0.0)	44 (0.1)
Total time in the ED, hours	7.6 [3.8–17.3]	11.8 [5.2–24.8]	20 [8.3–34]	10.4 [4.7–23]
In-hospital length, days	7 [4–14]	10 [5–19]	13 [7–24]	9 [4–18]
ICU admission	8,993 (31.6)	1,855 (30.1)	2,525 (19.4)	13,373 (28.1)
In-hospital mortality	1,121 (3.9)	496 (8.1)	1,527 (11.7)	3,144 (6.6)
Time between trauma and death, days	3.2 [1.2–9.2]	5 [1.5.2–15.7]	7.4 [2.7–17.5]	5.3 [1.9–14.3]

(One patient may have suffered from multiple injuries, the total of injuries presented in the table is greater than the number of patients.)

*ED* Emergency Department, *SBP* Systolic Blood Pressure, *ISS* Injury Severity Scale, *ICU* Intensive care unit

The most common severe injuries (AIS ≥ 3) affected the head in all age groups (27.9%, 35.6% and 29.9%, Online resource 1).

### Initial SBP and mortality

**Fig. 1:**
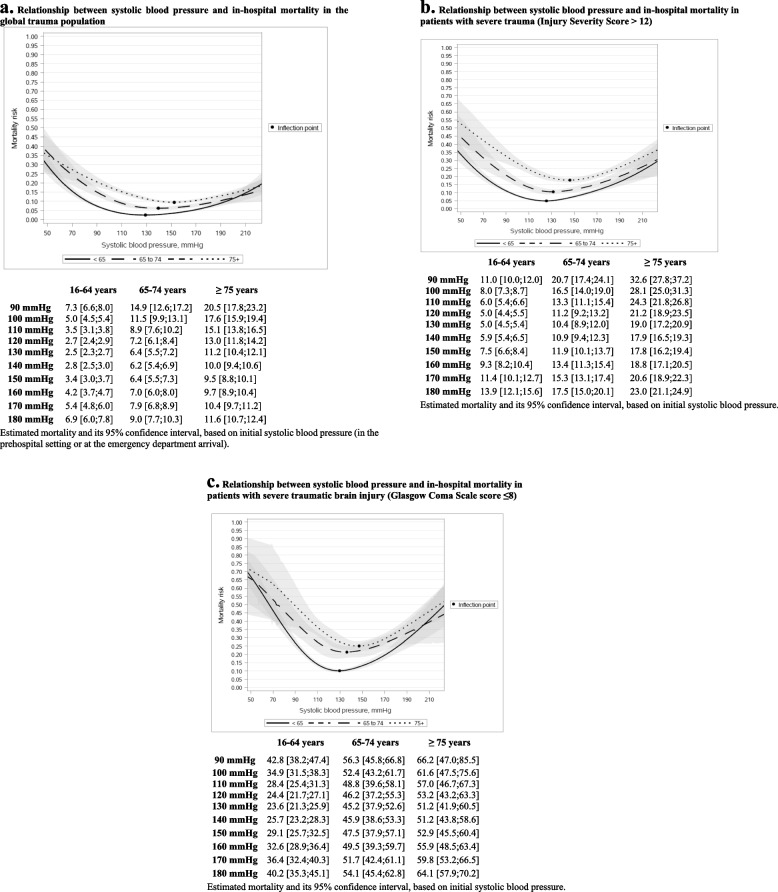
**a** Relationship between systolic blood pressure and in-hospital mortality in the global trauma population. **b** Relationship between systolic blood pressure and in-hospital mortality in patients with severe trauma (Injury Severity Score > 12). **c** Relationship between systolic blood pressure and in-hospital mortality in patients with severe traumatic brain injury (Glasgow Coma Scale score ≤ 8)

A nonlinear, U shape relationship between SBP and mortality was found in both older and younger patients. Mortality increased in all age groups across a wide range of SBP (from 130 to 90 mmHg), and this increase was higher for older patients (Fig. 1a). We observed distinct inflection points where mortality demonstrated an escalation in response to a decrease in SBP. Specifically, in the group of patients aged 16–64 years, the inflection point was identified at 129 mmHg while it was at 139 mmHg for individuals aged 65–74 years, and at 152 mmHg mmHg for patients aged ≥ 75 years (Fig. 1a). Similar results were observed with major trauma admissions (125, 131, and 146 mmHg respectively, Fig. 1b) and severe TBI (129, 130, and 135 mmHg respectively, Fig. 1c).

When assessing cohort effect in the three periods (2003–2007, 2008–2012 and 2013–2017), the results were similar in the global trauma population (Online resource 2a), as well as in the major trauma population (Online resource 2b) and the group of patients presenting with severe TBI (Online resource 2c).

**Fig. 2:**
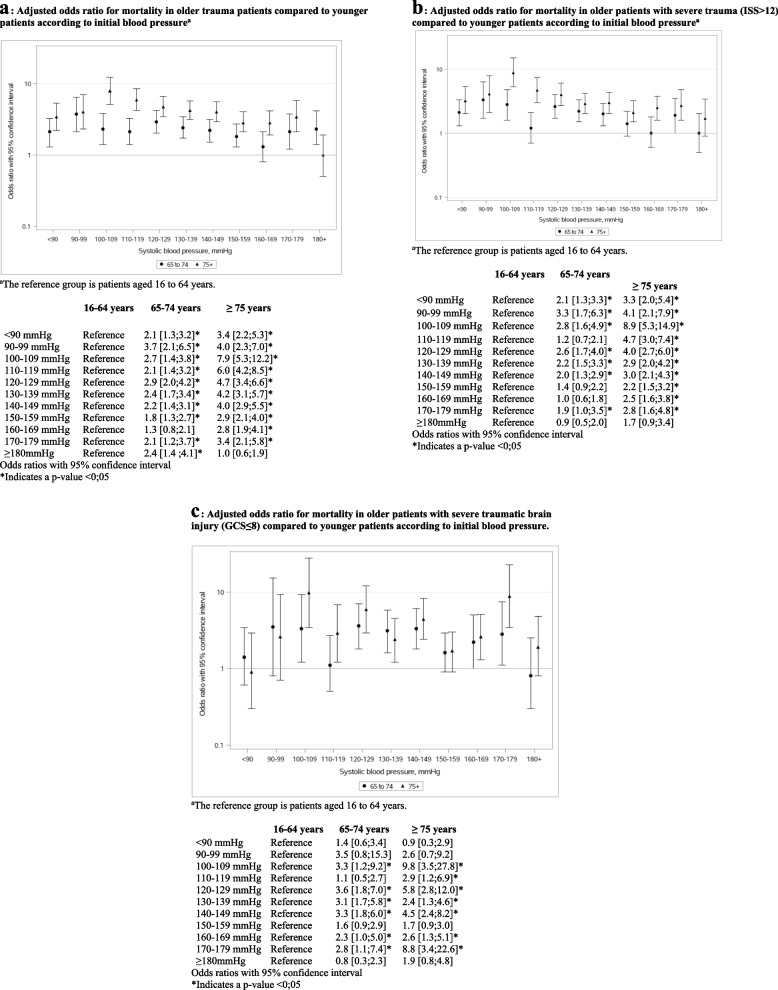
**a** Adjusted odds ratio for mortality in older trauma patients compared to younger patients according to initial blood pressure^a^. **b** Adjusted odds ratio for mortality in older patients with severe trauma (ISS > 12) compared to younger patients according to initial blood pressure^a^. **c** Adjusted odds ratio for mortality in older patients with severe traumatic brain injury (GCS ≤ 8) compared to younger patients according to initial blood pressure

Older age was associated with a higher odd for mortality at all ranges of SBP compared to younger adults (Fig. 2a). The highest OR for 65–74 years patients were 3.7 [95% CI: 2.1;6.5] in the 90–99 mmHg SBP range and 7.9 [95% CI: 5.1;12.2] for those aged ≥ 75 years in the 100–109 mmHg SBP range. Similar findings were found in the group of patients with major trauma (Fig. 2b) and in severe TBI patients with a SBP of 100–109, 120–129 and 130–139 mmHg (Fig. 2c).

A higher odd for mortality was also found in older adults with hypertension (140 mmHg to ≥ 180 mmHg) compared to their younger counterparts (Fig. 2a).

## Discussion

Our large Canadian trauma registry study found that the relationship between SBP and in-hospital mortality was non-linear (U-shape). Mortality was higher in older adults compared to their younger counterparts at all SBP points. It was also noted that hypotension was associated with a low sensitivity to predict mortality across all age groups when used alone.

### Interpretation of findings and comparison to previous studies

The American College of Surgeons Committee on Trauma (ACS-COT) recommends treating patients with severe trauma in level-I or II trauma centres. This includes adults with SBP < 90 mmHg at any time [28]. Most current prehospital algorithms use a 90 mmHg cutoff to identify severe trauma patients, including those based on Vittel triage criteria [29]. This cutoff has been widely debated, and some authors previously suggested that a SBP threshold of < 110 mmHg could be a more clinically accurate definition of hypotension [30–33]. Some even suggested integrating thresholds higher than 110 mmHg in triage protocols. For instance, Oyetunji et al. reported that the highest area under the receiver operating characteristic curve (AUC) curve value for patients aged ≥ 65 years intersected at an SBP of 117 mmHg [16]. Elsewhere a 120 mmHg threshold was found to better predict mortality for patients aged between 50 and 69 years compared to 140 mmHg for those aged ≥ 70 years [34].

Brown et al. compared the impact of two SBP thresholds (90 mmHg *vs* 110 mmHg) on older adults (≥ 65 years) triage performance. They reported that using the higher threshold of 110 mmHg led to a 4.4% reduction of undertriage. The authors have also found that the optimal SBP threshold to predict mortality would be 118 mmHg, yielding a sensitivity of 29% and a specificity of 86% [14]. Increasing the SBP threshold to redefine hypotension in older adults may lead to lower undertriage rates, a critical issue in that population, which was found to be at higher odd of mortality compared to their younger counterparts with equal or less severe injuries. However, despite improved performance to predict poor outcomes a cutoff of < 110 mmHg remained associated with low sensitivity in previous studies as in the present one [31–33]. Thus, this suggests that hypotension should not be used alone to predict mortality in trauma patients.

Hypertension was also associated with increased mortality in trauma patients. This may be related to multiple underlying mechanisms. First, hypertension may exacerbate hemorrhages, leading to worsened outcomes, justifying the concept of permissive hypotension [35]. Second, hypertension may be a marker of underlying cardiovascular comorbidities that increase the risk of mortality in patients with severe trauma. Patients with hypertension may also be prone to cardiovascular events, such as myocardial infarction, which can increase mortality in patients with severe trauma. Third, hypertension can result in alterations to the microcirculation that decrease blood flow to injured tissues, hence increasing the risk of complications. Reynolds et al. hypothesized in a rat model of hemorrhagic shock that systemic and microvascular alterations accompanying chronic hypertension would increase the vulnerability to hemorrhage relative to normotensive controls [36]. Fourth, hypertension may be the consequence of severe brain injuries resulting in intracranial hypertension (Cushing reflex) [37].

### Strengths and limitations

The present study is not without limitations. First, when prehospital SBP data was unavailable, we used initial SBP measured upon ED arrival, which may be slightly different. Nevertheless, paramedics were not trained to perform fluid resuscitation or to use vasopressors which could have led to a measurement variation between pre- and in-hospital SBP. Furthermore, in a similar prehospital system, Bruns et al. noted that the prehospital SBP was strongly correlated with the first SBP in the ED [30]. Another potential limitation is that only patients who met the RTQ inclusion criteria and were treated in level-I trauma centres were included in our analyses. Hence, our findings could be used to debate a new SBP threshold dedicated to identifying a major trauma or to activate a trauma team for older patients admitted to a level-I trauma centre. Finally, the odds ratio presented here likely overstate the true associations, given the elevated mortality risks observed among older patients [38].

The accuracy of the outcome (all causes of in-hospital mortality) could be debated. Indeed, some patients with non-trauma-related deaths may have been included in the analysis since the cause of death is not reported in the registry. Nevertheless, this potential bias is minor since half of the deaths occurred within the first five days after the trauma. It should also be acknowledged that some potential confounders that we were unable to consider and may impact the present findings.

Patient chronic conditions such as hypertension and anti-hypertensive medication usage may have affected SBP measures and should be considered in future investigations. In addition, some patients with TBI may experience high blood pressure due to intracranial hypertension (Cushing’s triad), which is associated with increased mortality risk. Therefore, the relationship between high blood pressure and mortality in TBI patients remains unclear, and further research is warranted to explore whether higher mortality rates are due to the severity of TBI-induced intracranial hypertension or HBP itself.

### Clinical implications

Higher SBP cutoffs should be considered to define hypotension in current triage protocols or trauma team activation criteria for patients over the age of 65. The study also highlights the U-shaped relationship between SBP and mortality among injured patients admitted to the ED. Consequently, healthcare providers should be aware that patients presenting with hypertension may also be at higher risk for mortality. As such, widely used binary cutoffs to define hypotension and predicting mortality should be abandoned in favor of more tailored approaches. Future digital tools should consider integrating SBP as a continuous rather than a dichotomized predictor to account for the U-shaped relationship between SBP and mortality. Such approach may contribute to mitigate undertriage in this older adult population.

Finally, the study highlights the limitations of using hypotension alone to predict mortality, given its low sensitivity.

### Research implications

This study was not designed to determine which SBP threshold would be optimal to predict mortality. This would require building or updating clinical decision rules with state-of-the-art methodology [39, 40]. Furthermore, as has been emphasized by other authors, continuous predictors (SBP in the present study) should not be dichotomized [41]. This may create problems rather than solving them, notably a considerable loss of information. Hence, further age-based risk prediction model studies are required to develop senior-friendly triage tools, in which other predictors should be integrated along with SBP to predict mortality. In addition, clinical decision tools should include SBP modelled using restricted cubic spines of fractional polynomials to capture the nonlinear association with in-hospital mortality.

## Conclusion

The relationship between SBP and mortality is nonlinear, regardless of trauma severity and age. Older age was associated with a higher odd of mortality at all SBP points. Future triage tools should therefore consider SBP as a continuous rather than a dichotomized predictor.

## Data Availability

The authors used governmental datasets (the Registre des traumatismes du Québec) for this study. These datasets are available through a formal request at the *Commission d’accès à l’information of the Gouvernement du Québec*.

## Abbreviations

SBP: Systolic blood pressure
mmHg: Millimetres of mercury
OR: Odds Ratio
CI: Confidence Interval
ED: Emergency Department
RTQ: Quebec Trauma Registry
IQR: Interquartile range
SD: Standard Deviation
ISS: Injury Severity Score
AIS: Abbreviated Injury Scale
GCS: Glasgow Coma Scale
TBI: Traumatic Brain Injury
SAS: Statistical Analysis System
ACS-COT: American College of Surgeons Committee on Trauma
AUC: Area Under the receiver operating characteristic curve
